# Compact Four-Port Metasurface for Tri-Band Operation in X-Band MIMO Applications

**DOI:** 10.3390/mi17070785

**Published:** 2026-06-27

**Authors:** Thamer S. Almoneef, Maged A. Aldhaeebi

**Affiliations:** Electrical Engineering Department, College of Engineering, Prince Sattam Bin Abdulaziz University, Al-Kharj 11942, Saudi Arabia; t.almoneef@psau.edu.sa

**Keywords:** metasurface, MIMO, X-band, gain, coupling, multibands

## Abstract

This paper presents the design, fabrication, and experimental validation of a compact four-port metasurface for tri-band X-band multiple-input multiple-output (MIMO) applications operating at 8.75 GHz, 9.75 GHz, and 10.5 GHz. The proposed structure employs a scalable unit-cell configuration to enhance radiation performance while maintaining a compact footprint. A four-port feeding mechanism is integrated to support MIMO operation with improved channel diversity and reduced mutual coupling. The metasurface is realized using a 32×32 unit-cell array, where increasing the number of unit cells significantly improves gain due to enhanced aperture efficiency. The fabricated prototype is experimentally characterized, and the measured S-parameters demonstrate good impedance matching at the three operating frequencies, with acceptable agreement between simulation and measurement results. In addition, reduced mutual coupling between ports confirms effective MIMO performance across the three bands. Radiation characteristics are evaluated through both 2D and 3D patterns. The radiation patterns were measured for a single port at frequencies where the reflection coefficient shows optimal performance, specifically at 8.75 GHz, 9.75 GHz, and 10.5 GHz. At these frequencies, the antenna exhibits well-defined main lobes with symmetrical radiation characteristics, indicating stable radiation behavior across the operating band. The realized gain exceeds 12 dBi at all three frequencies, with a peak gain of approximately 13 dB, along with satisfactory directivity and radiation efficiency. The results confirm that array scaling is an effective approach for gain enhancement without significantly increasing system complexity. In addition, the proposed MIMO metasurface achieves excellent diversity performance with ECC values below 0.04, DG values close to 10 dB, balanced MEG characteristics, and CCL values below 4 bits/s/Hz. The obtained results confirm that the proposed metasurface is a promising candidate for compact high-performance X-band MIMO systems for radar and advanced wireless communication applications.

## 1. Introduction

Metasurfaces have emerged as a powerful platform for controlling electromagnetic (EM) waves with subwavelength resolution, enabling compact and high-performance antenna systems for modern wireless applications [1,2,3]. By engineering the geometry of subwavelength unit cells, metasurfaces can manipulate amplitude, phase, and polarization of EM waves, offering unprecedented flexibility compared to conventional structures. In particular, metasurface-based antennas have gained significant attention in X-band systems due to their ability to achieve a high gain, a low profile, and flexible radiation control [4,5,6]. These features make them highly suitable for radar, satellite communication, and sensing applications.

Recently, multiple-input multiple-output (MIMO) technology has become essential in wireless communication systems to improve channel capacity, link reliability, and spectral efficiency [7,8,9]. However, integrating MIMO functionality into compact antenna structures remains challenging due to increased mutual coupling and limited available aperture [10,11,12]. High mutual coupling degrades diversity performance and reduces system efficiency, making it a critical issue in practical implementations. To mitigate these challenges, various techniques have been proposed, including defected ground structures (DGSs), decoupling networks, parasitic elements, and electromagnetic bandgap (EBG) structures [13,14,15]. Despite these efforts, achieving both compactness and high gain in MIMO metasurface systems remains an open research problem.

In this context, metasurface arrays provide a promising solution by enabling gain enhancement through aperture scaling [16,17,18]. By increasing the number of unit cells, the effective radiating aperture is enlarged, which directly improves antenna directivity and radiation efficiency. This concept is particularly attractive for high-frequency applications such as the X-band, where compact and high-performance antennas are required. However, the design must be carefully optimized to maintain a balance between performance improvement, structural complexity, and fabrication feasibility.

In this work, a compact four-port metasurface for X-band MIMO applications is proposed and experimentally validated. The design is based on a scalable unit-cell architecture, where gain enhancement is achieved by increasing the array size to a 32×32 configuration. A four-port feeding mechanism is integrated into the metasurface to support MIMO operation with improved channel diversity and acceptable isolation between ports. The proposed structure is fabricated and experimentally characterized. Both simulated and measured results are presented, demonstrating good agreement in S-parameters and radiation characteristics. The results show that the proposed metasurface achieves directional radiation with enhanced gain while maintaining compact dimensions. The findings confirm that scalable array design is an effective method for gain enhancement in compact metasurface-based MIMO systems, making the proposed structure a strong candidate for X-band radar and wireless communication applications.

## 2. Metasurface and Antenna Design

**Figure 1:**
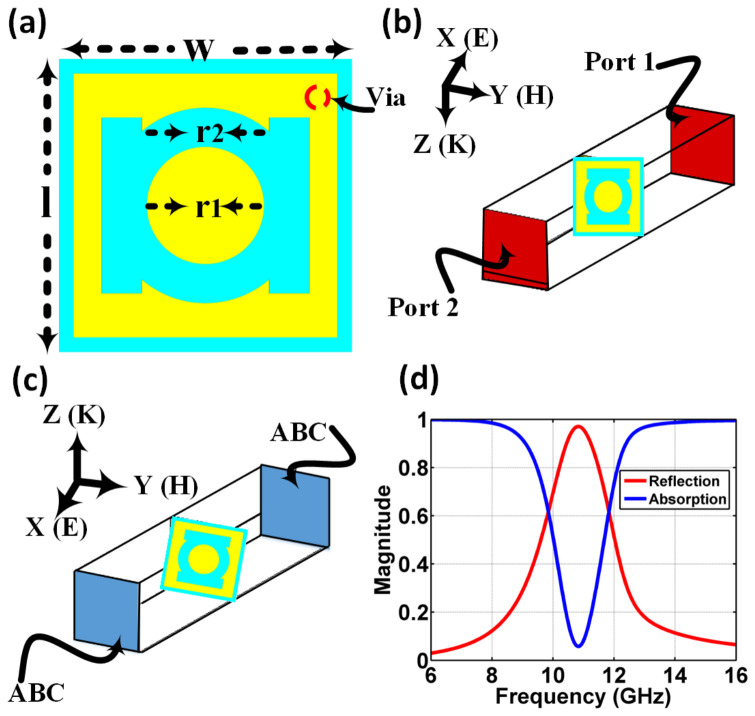
Geometry and simulation setup of the proposed metasurface unit cell for tri-band X-band MIMO applications: (**a**) unit-cell structure and design parameters, (**b**) CST waveguide-port configuration for reflection analysis, (**c**) boundary-condition setup for electromagnetic characterization, and (**d**) simulated reflection and absorption responses of the proposed structure.

The proposed metasurface unit cell features a compact symmetric geometry designed for tri-band X-band MIMO applications. As shown in Figure 1a, the structure consists of a square metallic loop integrated with two half-cylinder sections of radius $r_{2}=1.7\;\mathrm{mm}$ and a central circular resonator with radius $r_{1}=1.6\;\mathrm{mm}$. The interaction between these elements enhances current distribution and field confinement, enabling multiple resonances at 8.75 GHz, 9.75 GHz, and 10.5 GHz.

The unit cell is fabricated on a Rogers RO4003C substrate with thickness h=0.8mm, dielectric constant $\varepsilon_{r}=3.55$, and loss tangent tanδ=0.0027. The overall dimensions are w=l=4mm with a via gap of g=0.4mm and copper thickness t=35μm. A shunt-fed port with impedance $Z_{0}=200\;\Omega$ connects the top and bottom metallic layers through a via for efficient excitation.

Figure 1b,c illustrate the CST simulation setup and boundary conditions used for electromagnetic analysis. Periodic boundaries were applied to emulate an infinite metasurface array. The simulated reflection and absorption responses shown in Figure 1d demonstrate strong resonance behavior within the X-band region. When integrated into a large array, the coupling between adjacent unit cells improves aperture efficiency, realized gain, and overall MIMO performance.

The proposed metasurface antenna is designed to operate at three distinct frequencies within the X-band spectrum, specifically at 8.75 GHz, 9.75 GHz, and 10.5 GHz. The overall structure consists of a compact four-port metasurface array implemented using a scalable unit-cell configuration to enhance the radiation performance while maintaining a low-profile geometry. The metasurface is realized using a periodic arrangement of 32×32 unit cells printed on a dielectric substrate. The total dimensions of the proposed structure are 138mm×138mm. Each unit cell is carefully optimized to provide suitable electromagnetic resonance characteristics across the desired operating frequencies. The periodic arrangement increases the effective aperture area, which consequently improves the antenna gain and directivity. The antenna structure consists of two metallic layers. The top layer contains the metasurface radiating elements, while the bottom layer incorporates the feeding network responsible for exciting the four ports independently. The feeding structure is designed to provide balanced excitation with acceptable impedance matching and reduced mutual coupling between adjacent ports. The proposed four-port configuration enables MIMO operation, which improves channel diversity and enhances communication reliability. The spatial arrangement of the ports is optimized to minimize surface-wave interaction and reduce coupling effects between the radiating elements.

**Figure 2:**
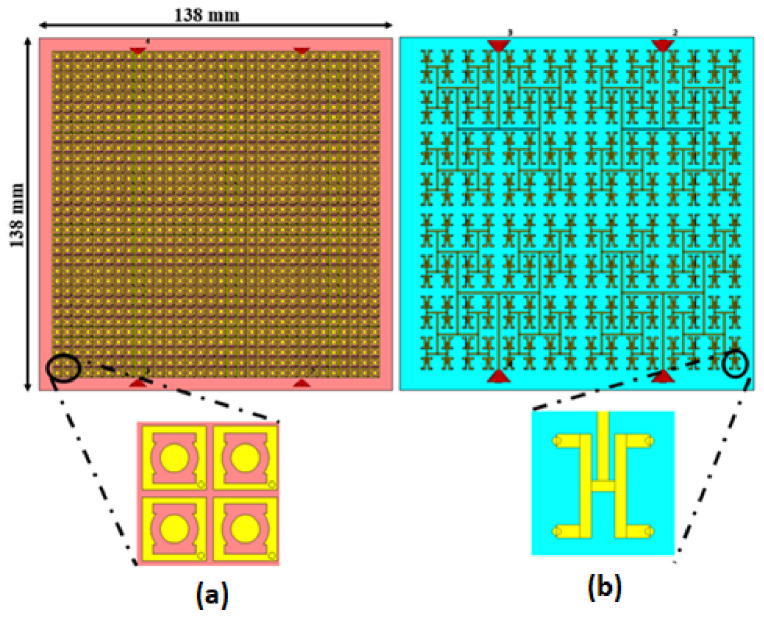
Geometry of the proposed four-port metasurface consisting of a 32×32 unit-cell array with overall dimensions of 138mm×138mm, including detailed unit-cell configurations: (**a**) top view of the proposed metasurface and (**b**) bottom view illustrating the feeding network.

The proposed design achieves tri-band operation due to the combined effect of the metasurface geometry and the coupling between neighboring unit cells. In addition, the large array aperture significantly enhances the radiation performance and improves the overall gain of the antenna system. Figure 2 illustrates the geometry of the proposed four-port metasurface, including the top and bottom views of the antenna configuration.

## 3. Fabrication and Measurement

**Figure 3:**
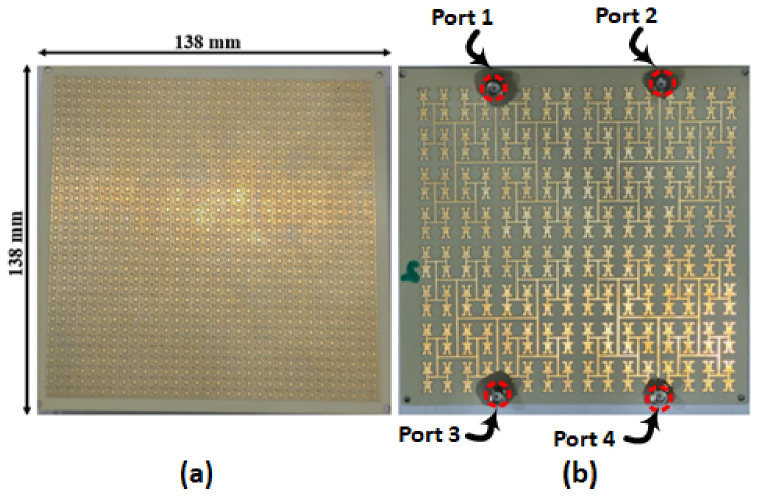
Fabricated prototype of the proposed four-port metasurface antenna showing the realized 32×32 unit-cell array: (**a**) top view of the fabricated metasurface and (**b**) bottom view showing the feeding network configuration.

The proposed metasurface antenna was fabricated using standard printed circuit board (PCB) technology as shown in Figure 3. The radiating structure and feeding network were etched on a dielectric substrate with compact dimensions suitable for X-band applications. SMA connectors were integrated into the four feeding ports to facilitate experimental characterization.

The fabricated prototype was experimentally evaluated using a vector network analyzer (VNA) to measure the scattering parameters of the antenna. During the measurements, one port was excited while the remaining ports were terminated with matched 50Ω loads to ensure accurate characterization of the reflection and coupling coefficients.

The reflection coefficients ($S_{11}$, $S_{22}$, $S_{33}$, and $S_{44}$) were measured across the X-band frequency range to validate the impedance matching performance of the antenna. In addition, the mutual coupling parameters between all antenna ports were measured to evaluate the MIMO isolation characteristics.

**Figure 4:**
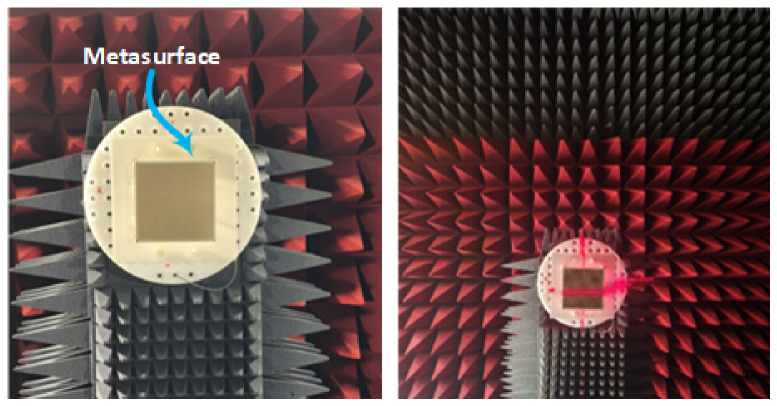
Experimental measurement setup used for characterizing the fabricated four-port metasurface antenna, including the vector network analyzer and far-field radiation measurement arrangement inside the anechoic chamber.

Radiation characteristics including gain, directivity, and radiation patterns were measured inside an anechoic chamber as shown in Figure 4. The measurements were conducted at the three operating frequencies of 8.75 GHz, 9.75 GHz, and 10.5 GHz. The antenna under test was mounted on a rotating platform to obtain the far-field radiation patterns in both principal planes.

The fabricated prototype demonstrates good agreement between simulated and measured results, confirming the validity of the proposed design methodology and the effectiveness of the metasurface configuration for high-gain MIMO applications.

## 4. Results and Discussion

**Figure 5:**
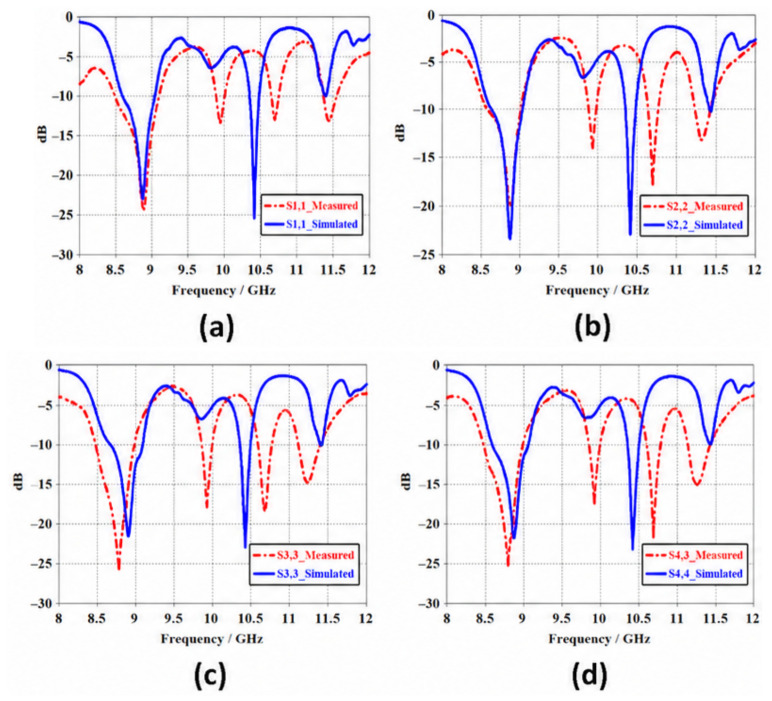
Simulated and measured S-parameters of the proposed four-port metasurface across the X-band, demonstrating satisfactory performance characteristics: (**a**) $S_{11}$, (**b**) $S_{22}$, (**c**) $S_{33}$, and (**d**) $S_{44}$.

The simulated and measured reflection coefficients of the proposed four-port metasurface antenna are presented in Figure 5. The results demonstrate good impedance matching at the three operating frequencies of 8.75 GHz, 9.75 GHz, and 10.5 GHz. A close agreement between the simulated and measured responses is observed for all ports, confirming the accuracy of the proposed design and fabrication process. Small deviations between the results are mainly attributed to fabrication tolerances, connector losses, and measurement uncertainties.

**Figure 6:**
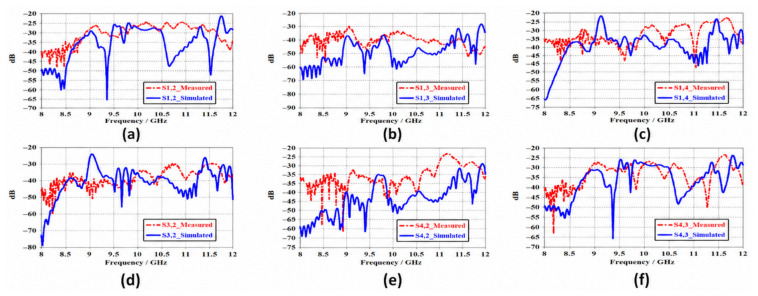
Simulated and measured mutual coupling coefficients of the proposed four-port metasurface across the X-band, indicating satisfactory port isolation performance: (**a**) $S_{12}$, (**b**) $S_{13}$, (**c**) $S_{14}$, (**d**) $S_{32}$, (**e**) $S_{42}$, and (**f**) $S_{43}$.

Figure 6 shows the simulated and measured mutual coupling coefficients between the antenna ports. The proposed structure achieves satisfactory isolation performance across the operating bands due to the optimized port arrangement and metasurface configuration. The coupling levels remain sufficiently low, indicating reduced electromagnetic interaction between adjacent ports and confirming the suitability of the antenna for MIMO applications.

**Figure 7:**
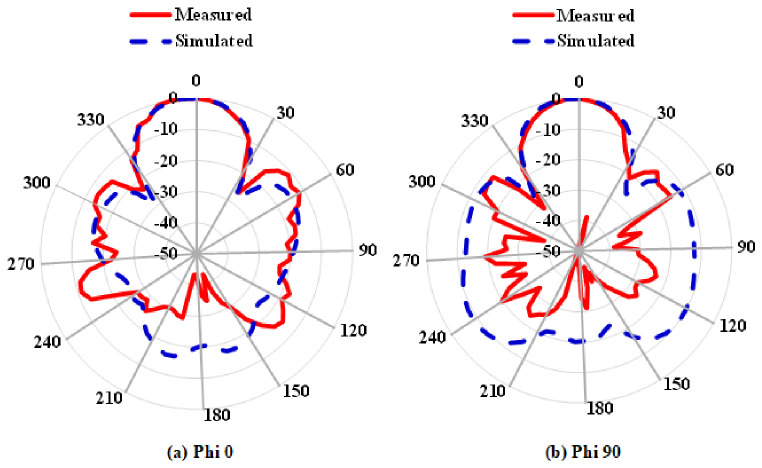
Simulated and measured 2D radiation patterns of the proposed four-port metasurface antenna at 8.75 GHz in the principal planes.

**Figure 8:**
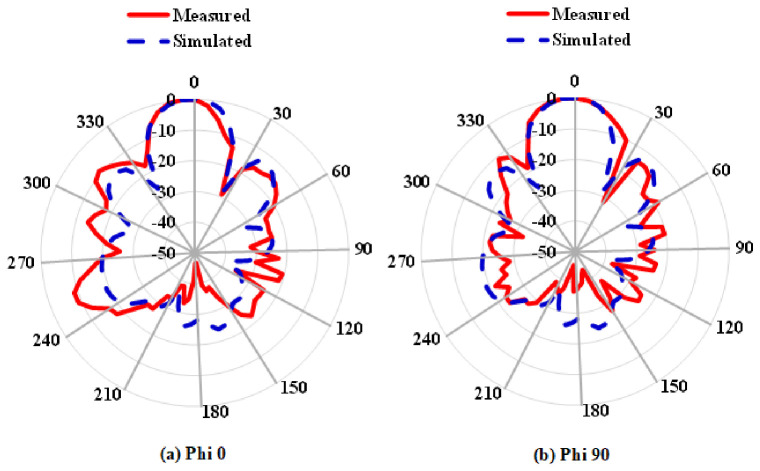
Simulated and measured 2D radiation patterns of the proposed four-port metasurface antenna at 9.75 GHz in the principal planes.

**Figure 9:**
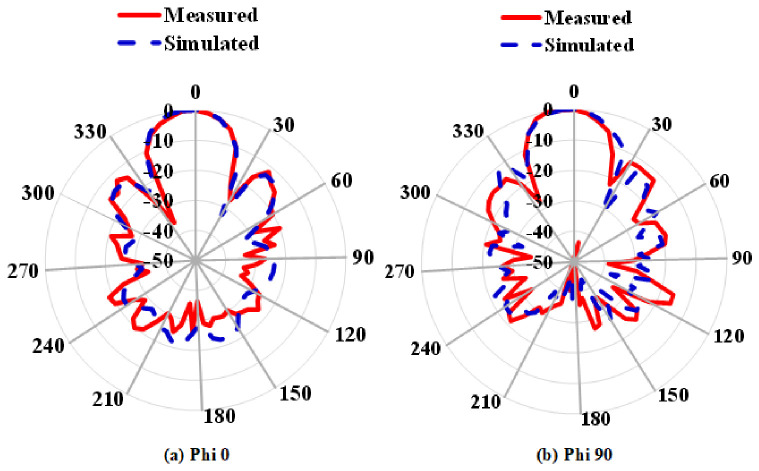
Simulated and measured 2D radiation patterns of the proposed four-port metasurface antenna at 10.5 GHz in the principal planes.

The radiation characteristics of the proposed metasurface antenna were evaluated through both simulated and measured radiation patterns. Figure 7, Figure 8 and Figure 9 illustrate the 2D radiation patterns at 8.75 GHz, 9.75 GHz, and 10.5 GHz, respectively. The antenna exhibits stable directional radiation with symmetrical main lobes and relatively low side-lobe levels across the three operating frequencies. The measured results show acceptable agreement with the simulated patterns, validating the effectiveness of the metasurface array design.

**Figure 10:**
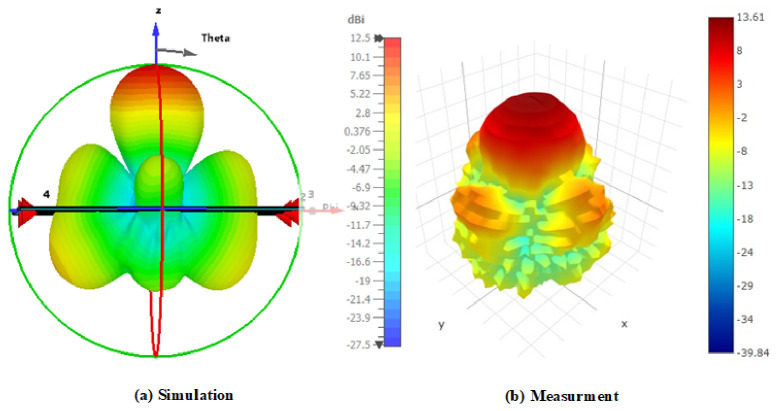
Simulated 3D radiation pattern and realized gain distribution of the proposed metasurface antenna at 8.75 GHz.

**Figure 11:**
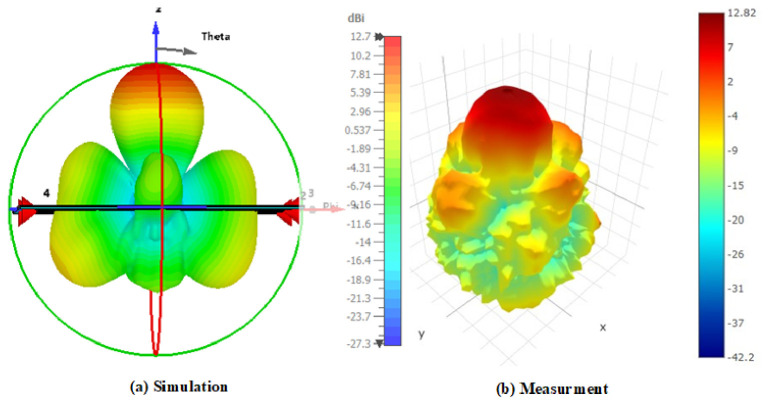
Simulated 3D radiation pattern and realized gain distribution of the proposed metasurface antenna at 9.75 GHz.

**Figure 12:**
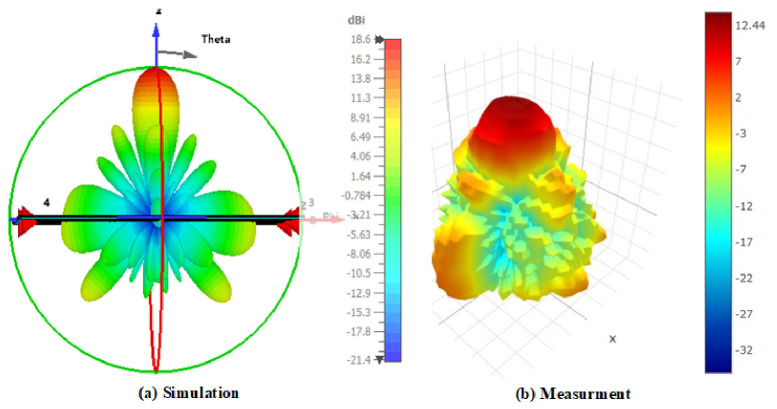
Simulated 3D radiation pattern and realized gain distribution of the proposed metasurface antenna at 10.5 GHz.

Figure 10, Figure 11 and Figure 12 present the corresponding 3D radiation patterns and gain performance of the proposed antenna. The large 32 × 32 metasurface array significantly improves the effective aperture area, resulting in enhanced directivity and realized gain. The antenna achieves realized gains exceeding 12 dBi at all operating frequencies, with a peak gain of approximately 13 dBi near 10.5 GHz. The enhanced gain performance confirms the effectiveness of the scalable metasurface configuration for X-band applications.

The envelope correlation coefficient (ECC) is an important parameter used to evaluate the diversity performance and mutual correlation between MIMO antenna elements. It can be calculated using the scattering parameters as follows:

(1) $$\mathrm{ECC}=\frac{{\left|S_{11}S_{12}^{*}+S_{21}S_{22}^{*}\right|}^{2}}{\left(1-|S_{11}{|}^{2}-{\left|S_{21}\right|}^{2}\right)\left(1-|S_{22}{|}^{2}-{\left|S_{12}\right|}^{2}\right)}$$

For good MIMO performance, the ECC value should be less than 0.5, while values below 0.1 indicate excellent diversity characteristics.

The mean effective gain (MEG) is an important MIMO performance parameter that evaluates the average received power of each antenna element in a multipath propagation environment. For an efficient MIMO system, the difference in MEG between antenna elements should remain within acceptable limits to ensure balanced power reception and uniform radiation behavior. The proposed four-port metasurface antenna demonstrates stable MEG characteristics across the operating frequency bands, with only minor variations between the antenna ports. The measured and simulated MEG results exhibit good agreement, confirming that the antenna elements possess balanced reception capability and suitable diversity performance for practical X-band MIMO communication systems. MEG describes the average received power performance of each antenna element in a multipath environment. For proper MIMO operation, the MEG difference between antenna elements should generally remain below 3 dB and is calculated as

(2) $$MEG_{i}=0.5\left(1-\sum_{j=1}^{N}{\left|S_{ij}\right|}^{2}\right)$$

The MEG balance condition is expressed as

(3) $$|MEG_{i}-MEG_{j}|\;<\;3\;\mathrm{dB}$$

The diversity gain (DG) indicates the capability of the MIMO antenna system to mitigate multipath fading effects. A DG value close to 10 dB is considered desirable for efficient diversity performance. The DG is determined from the ECC values using

(4) $$DG=10\sqrt{1-{\left|ECC\right|}^{2}}$$

The channel capacity loss (CCL) quantifies the reduction in channel capacity caused by mutual coupling and correlation effects between antenna elements. Lower CCL values indicate better MIMO system performance. The CCL is calculated using

(5) $$CCL=-\log_{2}\left(\det(\Psi^{R})\right)$$

where the correlation matrix is given by

(6) $$\Psi^{R}=\left[\begin{array}{cc} \rho_{11} & \rho_{12}\\ \rho_{21} & \rho_{22} \end{array}\right]$$

The matrix elements are expressed as

(7) $$\rho_{ii}=1-\sum_{n=1}^{N}{\left|S_{in}\right|}^{2}$$

and

(8) $$\rho_{ij}=-\left(S_{ii}^{*}S_{ij}+S_{ji}^{*}S_{jj}\right)$$

**Figure 13:**
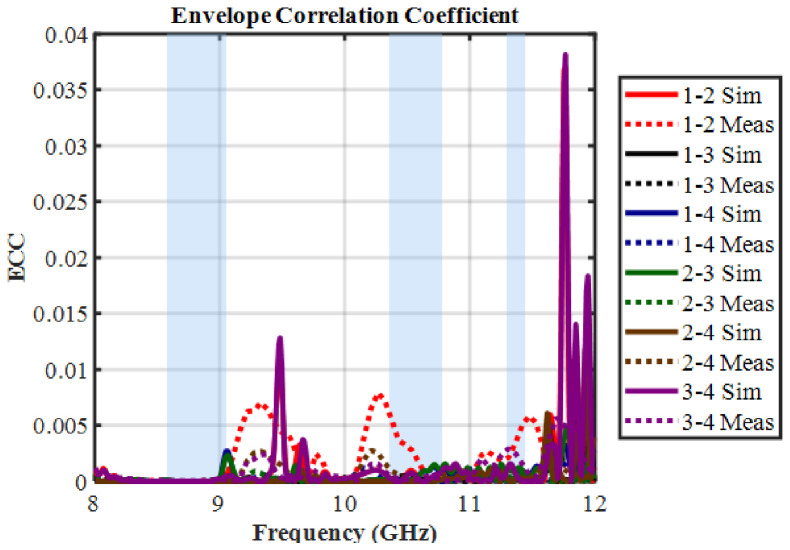
Simulated and measured envelope correlation coefficient (ECC) of the proposed four-port tri-band metasurface MIMO antenna.

Figure 13 illustrates the simulated and measured envelope correlation coefficient (ECC) for different port combinations of the proposed four-port metasurface MIMO antenna. The obtained ECC values remain below 0.04 throughout the operating frequency range, which is significantly lower than the acceptable threshold of 0.5 for practical MIMO systems. Most port combinations exhibit ECC values close to zero across the three operating bands, indicating excellent isolation and low correlation between antenna elements. Small deviations between simulation and measurement results are observed at higher frequencies, particularly near 11.7 GHz, which can be attributed to fabrication tolerances, connector losses, and measurement uncertainties. Nevertheless, the measured results closely follow the simulated trends, confirming the effectiveness of the proposed metasurface structure in achieving superior diversity performance and efficient MIMO operation.

**Figure 14:**
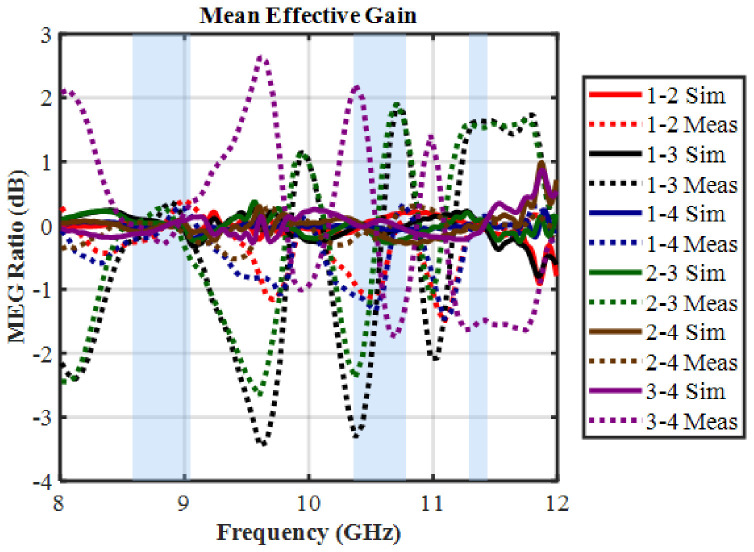
Simulated and measured mean effective gain (MEG) of the proposed four-port tri-band metasurface MIMO antenna.

Figure 14 presents the simulated and measured mean effective gain (MEG) characteristics for all antenna port combinations. The MEG values remain within the acceptable range, with variations between antenna elements less than 3 dB across the operating bands. This indicates a balanced power reception capability and uniform radiation performance among the antenna elements. The simulated MEG curves are generally close to 0 dB, while the measured results show slight fluctuations due to practical fabrication and measurement conditions. Despite these minor variations, the measured and simulated results demonstrate good agreement, confirming stable and efficient MIMO performance for the proposed metasurface antenna.

**Figure 15:**
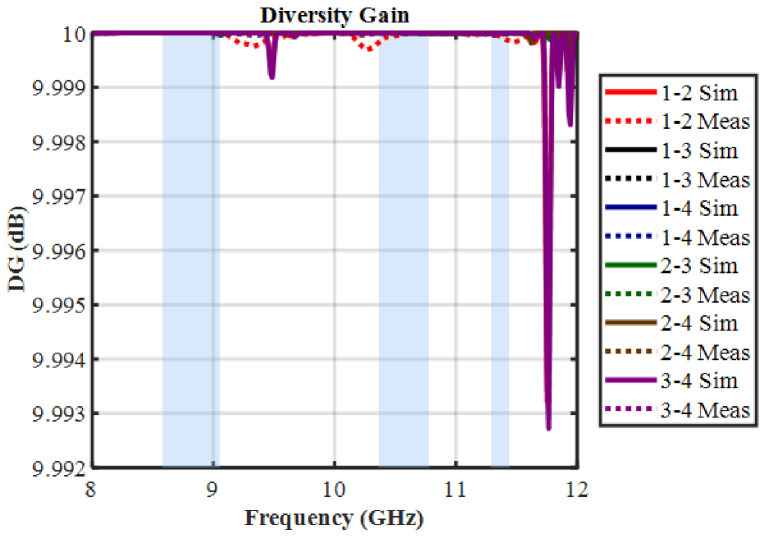
Simulated and measured diversity gain (DG) of the proposed four-port tri-band metasurface MIMO antenna.

Figure 15 shows the diversity gain (DG) performance of the proposed four-port metasurface antenna. The DG values remain approximately 10 dB across the entire operating frequency range for both simulated and measured results. Such high DG values indicate excellent diversity performance and effective mitigation of multipath fading effects in wireless communication environments. Slight reductions in the measured DG are observed at higher frequencies due to increased mutual coupling and practical measurement limitations. However, the overall agreement between simulation and measurement validates the capability of the proposed antenna to provide reliable diversity characteristics suitable for high-performance X-band MIMO systems.

**Figure 16:**
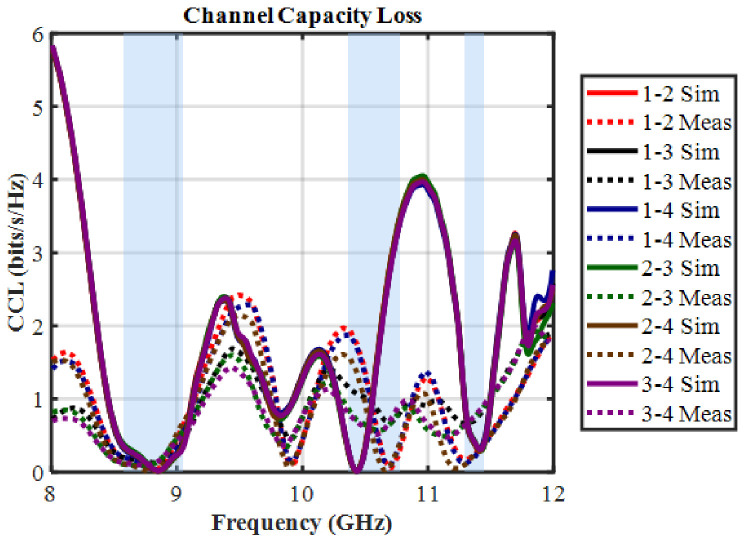
Simulated and measured channel capacity loss (CCL) of the proposed four-port tri-band metasurface MIMO antenna.

Figure 16 illustrates the simulated and measured channel capacity loss (CCL) of the proposed four-port MIMO metasurface antenna. The CCL values remain below 4 bits/s/Hz over the operating frequency range, satisfying the acceptable criterion for practical MIMO applications. Low CCL values indicate minimal loss in channel capacity and efficient transmission performance in multipath communication environments. The simulated and measured results exhibit similar trends with minor discrepancies caused by fabrication tolerances, connector mismatches, and environmental effects during measurements. Overall, the low ECC, high DG, balanced MEG, and acceptable CCL collectively confirm the excellent MIMO diversity performance of the proposed tri-band metasurface antenna.

**Figure 17:**
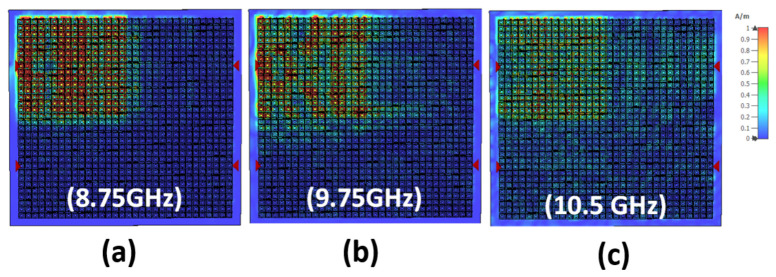
Simulated surface current distributions of the proposed metasurface antenna under Port-1 excitation at (**a**) 8.75 GHz, (**b**) 9.75 GHz, and (**c**) 10.5 GHz. The results reveal the dominant resonant modes associated with the outer square loop, the coupled outer-loop/semi-circular resonator, and the central circular resonator, respectively.

Although the proposed metasurface antenna exhibits relatively narrow operating bandwidths, this behavior is attributed to the high-*Q* resonant characteristics of the electrically small metasurface unit cells and the collective resonant modes generated by the finite 32×32 array. The surface current distributions shown in Figure 17 reveal strong electromagnetic field confinement at the three operating frequencies (8.75 GHz, 9.75 GHz, and 10.5 GHz), where different resonant elements contribute to the radiation mechanism. While this localized resonance improves aperture efficiency, realized gain, and MIMO performance, it naturally limits the achievable impedance bandwidth.

The design is mainly optimized to achieve distinct tri-band operation with high port isolation and stable MIMO characteristics rather than maximizing impedance bandwidth. The narrow resonances provide selective frequency channels, which can be beneficial for multi-band X-band communication applications by reducing interference between adjacent operating bands. Nevertheless, the obtained bandwidths are sufficient for several narrowband X-band applications, including radar, satellite communications, and frequency-selective wireless systems. Therefore, the proposed design provides an effective trade-off between bandwidth, gain enhancement, compactness, and MIMO diversity performance.

Surface current distributions were added and are presented in Figure 17. The current distributions provide physical insight into the origin of each resonant mode. At 8.75 GHz, the strongest current concentration is observed along the outer square loop, indicating that the lower resonance is mainly generated by the longest current path associated with the outer ring. At 9.75 GHz, the current is distributed over both the outer square loop and the semi-circular resonator, demonstrating a coupled resonance mechanism. At 10.5 GHz, the current becomes more concentrated around the central circular resonator and neighboring unit cells, confirming that the higher-frequency resonance is associated with the inner resonating structure and inter-element coupling.

**Figure 18:**
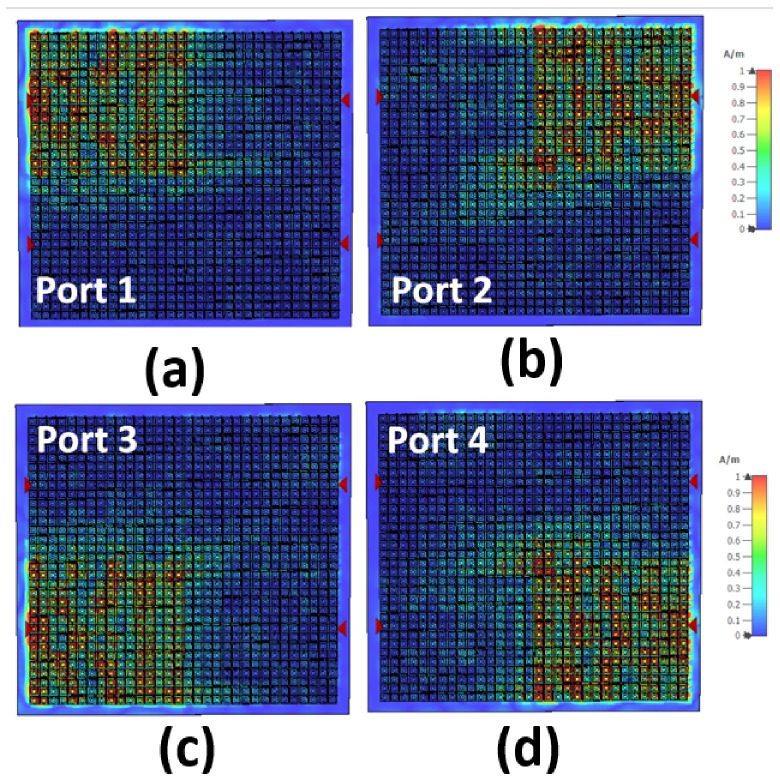
Simulated surface current distributions of the proposed four-port metasurface antenna when exciting (**a**) Port 1, (**b**) Port 2, (**c**) Port 3, and (**d**) Port 4. The electromagnetic energy is primarily confined around the excited region, confirming reduced mutual coupling and effective MIMO isolation.

Furthermore, the current distributions for the four excitation ports were also included, as shown in Figure 18. The results demonstrate that the electromagnetic energy remains primarily confined around the excited quadrant of the metasurface, while relatively weak currents are induced in the remaining regions. This behavior confirms the effectiveness of the proposed structure in suppressing mutual coupling and maintaining good isolation between the MIMO ports.

The surface current distributions shown in Figure 18 clearly identify the physical origin of the three resonant modes. At 8.75 GHz, the current is concentrated mainly along the outer square loop, producing the lowest resonant frequency due to the longest current path. At 9.75 GHz, the current is shared between the outer square loop and the semi-circular resonator, indicating a coupled resonant mode. At 10.5 GHz, the current becomes concentrated around the central circular resonator and neighboring cells, generating the highest-order resonance. These observations are in good agreement with the proposed equivalent LC circuit model and confirm the tri-band operating mechanism of the metasurface antenna.

**Figure 19:**
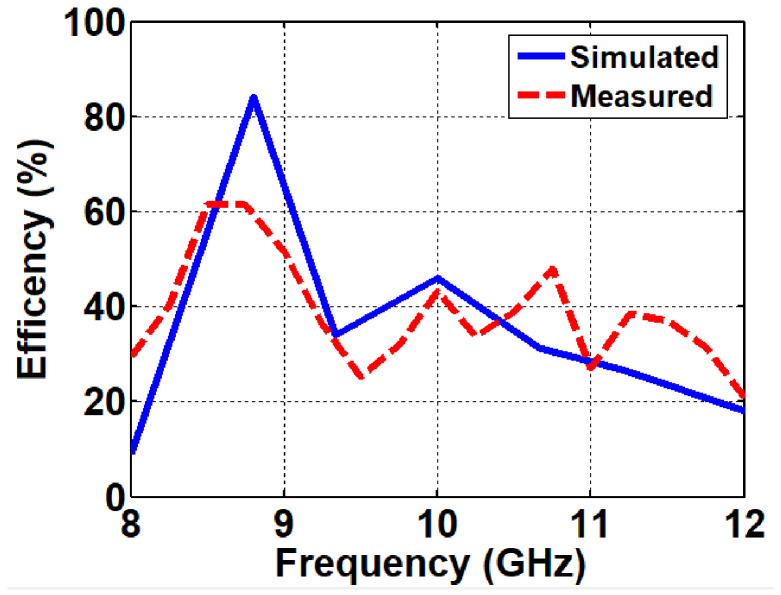
Simulated and measured total efficiency of the proposed four-port tri-band metasurface antenna across the X-band frequency range.

As shown in Figure 19, the total efficiency exhibits distinct peaks near the three operating frequencies of 8.75 GHz, 9.75 GHz, and 10.5 GHz. The highest efficiency is observed around 8.75 GHz, while additional efficiency maxima occur near 9.75 GHz and 10.5 GHz, corresponding to the three resonant modes supported by the proposed metasurface structure. The efficiency decreases away from these resonances due to increased impedance mismatch and reduced radiation effectiveness. Furthermore, the measured and simulated efficiency results show good agreement, confirming the validity of the proposed design and fabrication process.

**Figure 20:**
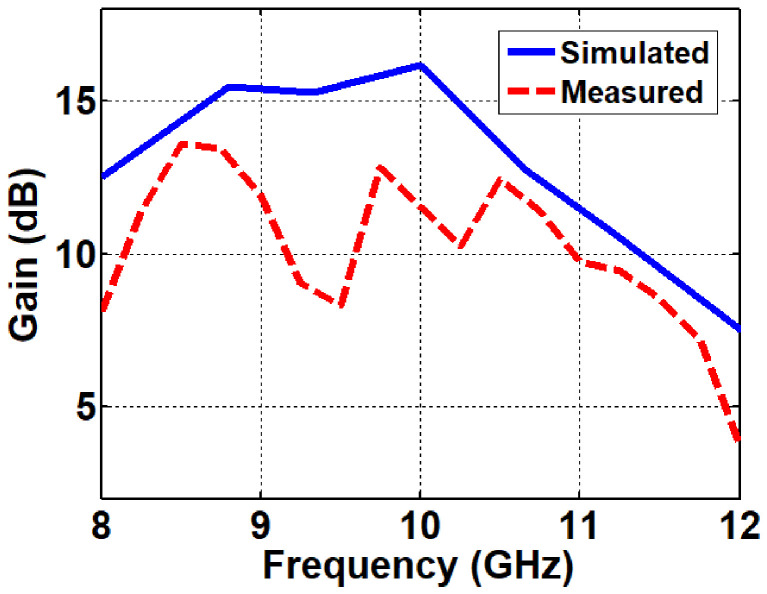
Simulated and measured realized gain of the proposed four-port tri-band metasurface antenna as a function of frequency across the operating band.

The realized gain curve, as shown in Figure 20, exhibits a similar trend, with gain enhancement occurring near the three resonant frequencies. The antenna achieves realized gains exceeding 12 dBi throughout the operating bands, with a peak gain approaching 13 dBi near 10.5 GHz. This improvement is mainly attributed to the large effective aperture provided by the 32×32 metasurface array, which enhances aperture efficiency and radiation directivity while maintaining a compact profile.

The combined efficiency and gain characteristics demonstrate that the proposed antenna effectively converts the input power into radiated electromagnetic energy at the three designed resonant frequencies of 8.75 GHz, 9.75 GHz, and 10.5 GHz while maintaining stable radiation performance. These results further confirm that the proposed metasurface antenna achieves an effective balance among compact size, high realized gain, acceptable efficiency, and excellent MIMO diversity performance, making it suitable for X-band radar and wireless communication applications.

Overall, the measured and simulated results confirm that the proposed metasurface antenna successfully achieves compact tri-band operation with high gain, stable radiation characteristics, and low mutual coupling. These features make the antenna a strong candidate for compact X-band MIMO systems, including radar and advanced wireless communication applications.

**Figure 21:**
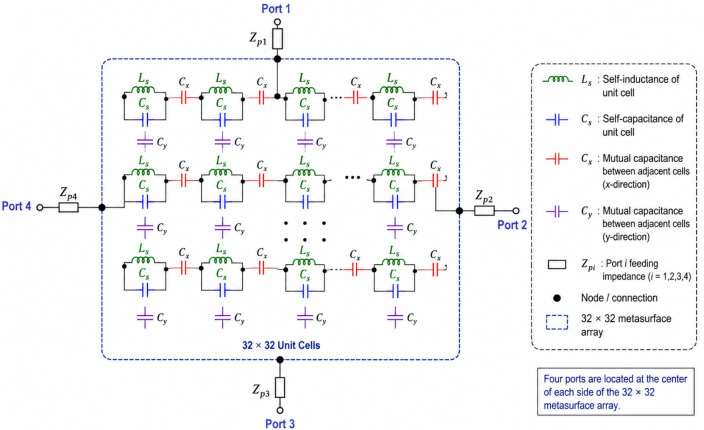
Equivalent circuit model of the proposed 32×32 metasurface array. Each unit cell is modeled as an LC resonator with inductance $L_{s}$ and capacitance $C_{s}$, while the inter-cell coupling is represented by mutual capacitance $C_{c}$. The observed tri-band response results from collective resonant modes due to the periodic array and feed–array interaction.

The isolated unit cell exhibits a single LC resonance determined by $L_{s}$ and $C_{s}$. However, when 32×32 unit cells are periodically arranged, strong electromagnetic interactions between neighboring cells introduce additional coupling capacitances ($C_{x}$ and $C_{y}$). These coupling effects result in resonance splitting and the formation of multiple collective modes. Consequently, the complete metasurface array exhibits three distinct resonant frequencies, whereas the individual unit cell supports only one fundamental resonance. The equivalent circuit model of the proposed 32×32 four-port metasurface array is illustrated in Figure 21.

The equivalent circuit model of the proposed metasurface is shown in Figure 21. The individual unit cell is modeled by a parallel LC resonator, where the metallic current paths contribute to the equivalent inductance L, while the gaps between metallic sections contribute to the equivalent capacitance C. The grounding via further increases the effective inductance of the resonant structure.

It should be noted that the isolated unit cell does not generate the three operating frequencies observed in the proposed antenna. Instead, the tri-band response emerges when the unit cells are arranged into the 32×32 metasurface array and excited through the four-port feeding structure. In the array configuration, strong electromagnetic coupling is established between adjacent cells through mutual capacitances and inductances, resulting in the formation of multiple collective resonant modes. Furthermore, the interaction between the feeding network and the finite metasurface aperture introduces additional resonant effects.

Consequently, the three resonances observed at 8.75 GHz, 9.75 GHz, and 10.5 GHz are attributed to the combined effects of the fundamental unit-cell resonance, inter-element coupling within the periodic array, and feed-induced array modes. This behavior is consistent with the surface-current distributions shown in Figure 18, where different current distributions are observed at each operating frequency, indicating the excitation of distinct array modes rather than independent resonances of a single unit cell.

Each metasurface unit cell is represented by an LC resonator consisting of self-inductance ($L_{s}$) and self-capacitance ($C_{s}$), while the electromagnetic coupling between adjacent cells is modeled using mutual capacitances ($C_{x}$ and $C_{y}$) along the horizontal and vertical directions, respectively. The four excitation ports are located at the centers of the four sides of the array and are represented by the feeding impedances $Z_{p1}$, $Z_{p2}$, $Z_{p3}$, and $Z_{p4}$.

**Figure 22:**
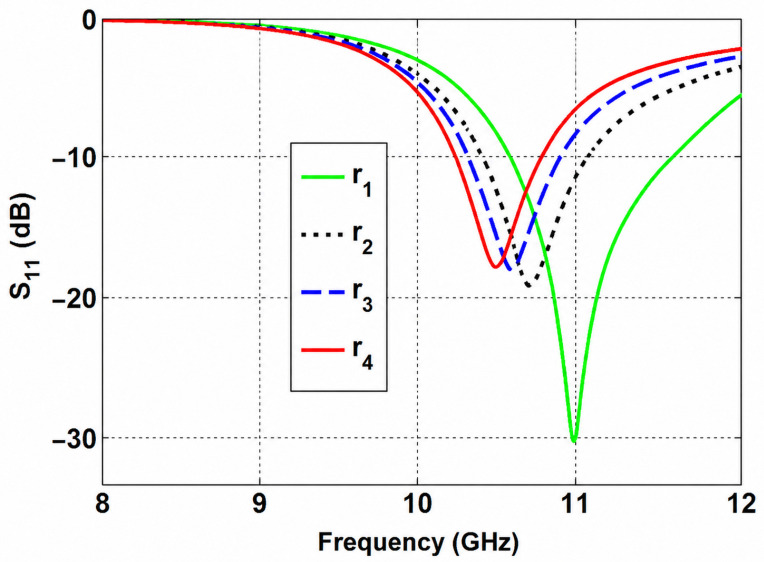
Parametric analysis showing the $S_{11}$ response of the unit-cell geometry by varying the central patch radius $r_{1}$ and half-cylinder radius $r_{2}$ with respect to the unit-cell length *L*.

The collective interaction of the coupled resonators across the 32×32 array gives rise to the observed tri-band resonant behavior and inter-port coupling characteristics, as shown in Figure 22.

The impedance of an individual metasurface unit cell can be represented by a parallel LC resonator as

(9) $$Z_{\mathrm{uc}}={\left(j\omega C_{s}+\frac{1}{j\omega L_{s}}\right)}^{-1}$$

where $L_{s}$ and $C_{s}$ denote the self-inductance and self-capacitance of the unit cell, respectively.

The resonance frequency of the isolated unit cell is given by:

(10) $$f_{0}=\frac{1}{2\pi \sqrt{L_{s}C_{s}}}$$

For the complete metasurface array, the electromagnetic coupling between adjacent unit cells is represented by the mutual capacitances $C_{x}$ and $C_{y}$ in the horizontal and vertical directions, respectively. The effective capacitance can therefore be expressed as:

(11) $$C_{\mathrm{eff}}=C_{s}+C_{x}+C_{y}$$

Accordingly, the resonance frequency of the coupled metasurface array becomes

(12) $$f_{r}=\frac{1}{2\pi \sqrt{L_{s}C_{\mathrm{eff}}}}$$

Therefore, the resonance frequency can also be written as

(13) $$f_{r}=\frac{1}{2\pi \sqrt{L_{s}\left(C_{s}+C_{x}+C_{y}\right)}}$$

The input impedance observed at Port *i* is expressed as:

(14) $$Z_{\mathrm{in},i}=Z_{p_{i}}+Z_{\mathrm{array}}$$

where $Z_{p_{i}}$ denotes the feeding impedance of Port *i*, and $Z_{\mathrm{array}}$ represents the equivalent impedance of the metasurface array.

The mutual coupling between Ports *i* and *j* is quantified using the scattering parameters as

(15) $$S_{ij}=20\log_{10}\left|\frac{V_{j}}{V_{i}}\right|\;\left(i\neq j\right)$$

where $V_{i}$ and $V_{j}$ are the incident and coupled voltages at Ports *i* and *j*, respectively.

The tri-band operation of the proposed 32×32 metasurface array can be modeled through three coupled resonant modes given by:

(16) $$f_{1}=\frac{1}{2\pi \sqrt{L_{s}\left(C_{s}+C_{m1}\right)}}$$

(17) $$f_{2}=\frac{1}{2\pi \sqrt{L_{s}\left(C_{s}+C_{m2}\right)}}$$

(18) $$f_{3}=\frac{1}{2\pi \sqrt{L_{s}\left(C_{s}+C_{m3}\right)}}$$

where $C_{m1}$, $C_{m2}$, and $C_{m3}$ represent the effective coupling capacitances associated with the three collective resonant modes generated by the finite metasurface array.

Although the isolated unit cell exhibits only a single resonance described by Equation (10), the strong inter-element coupling introduced by the periodic 32×32 arrangement generates additional collective eigenmodes. These modes are represented by the effective coupling capacitances $C_{m1}$, $C_{m2}$, and $C_{m3}$, resulting in the observed tri-band response of the complete metasurface array. This phenomenon is commonly referred to as resonance splitting in coupled resonator networks.

The additional resonances are not produced by the isolated unit cell itself but arise from the collective electromagnetic interaction among the large number of coupled elements in the 32×32 metasurface configuration. These coupling capacitances modify the effective capacitance of the resonant system, resulting in multiple resonance frequencies. This phenomenon is commonly referred to as resonance splitting in coupled resonator networks, where the interaction between neighboring resonators creates multiple eigenmodes and leads to the observed tri-band response of the complete metasurface array.

A parametric sensitivity analysis was performed to investigate the effect of the geometrical parameters $r_{1}$ and $r_{2}$ on the resonance frequency of the metasurface unit cell, as shown in Figure 22. The parameter $r_{1}$ represents the radius of the central patch, while $r_{2}$ denotes the radius of the half-cylinder structure. These parameters control the capacitive and inductive behavior of the unit cell and consequently affect the resonance frequency. Furthermore, the variation in $r_{1}$ and $r_{2}$ modifies the electromagnetic coupling between adjacent unit cells, which is required to excite strong collective coupling modes in the complete metasurface array. The investigated values are normalized with respect to the unit-cell length *L*, where $r_{1}=L/3$, $r_{2}=L/3.5$, $r_{3}=L/3.75$, and $r_{4}=L/4=1$. The influence of these parameters on the resonance behavior is illustrated in Figure 22, demonstrating their importance in controlling the operating frequencies and optimizing the proposed metasurface antenna performance.

## 5. Conclusions

A compact four-port metasurface antenna for tri-band X-band MIMO applications has been proposed, fabricated, and experimentally validated. The design employs a scalable 32 × 32 unit-cell metasurface configuration to enhance radiation performance while maintaining compact dimensions. The proposed antenna successfully operates at 8.75 GHz, 9.75 GHz, and 10.5 GHz with satisfactory impedance matching and low mutual coupling between ports. Experimental results demonstrate good agreement with simulations in terms of S-parameters, isolation characteristics, and radiation performance. The antenna achieves realized gains greater than 12 dBi with stable directional radiation patterns across the operating frequencies. In addition, the low ECC values confirm excellent diversity performance suitable for MIMO communication systems. The results verify that increasing the metasurface array size effectively improves aperture efficiency and antenna gain without significantly increasing design complexity. Therefore, the proposed metasurface antenna represents a promising solution for compact high-performance X-band MIMO applications such as radar, sensing, and advanced wireless communication systems.

## Data Availability

The original contributions presented in this study are included in the article. Further inquiries can be directed to the corresponding author.

## References

[1] Holloway C.L., Kuester E.F., Gordon J.A., O’Hara J., Booth J., Smith D.R. (2012). An overview of the theory and applications of metasurfaces: The two-dimensional equivalents of metamaterials. IEEE Antennas Propag. Mag..

[2] Glybovski S.B., Tretyakov S.A., Belov P.A., Kivshar Y.S., Simovski C.R. (2016). Metasurfaces: From microwaves to visible. Phys. Rep..

[3] Yu N., Capasso F. (2014). Flat optics with designer metasurfaces. Nat. Mater..

[4] Pfeiffer C., Grbic A. (2013). Metamaterial Huygens’ surfaces: Tailoring wave fronts with reflectionless sheets. Phys. Rev. Lett..

[5] Chen H.-T., Taylor A.J., Yu N. (2016). A review of metasurfaces: Physics and applications. Rep. Prog. Phys..

[6] Cui T.J., Qi M.Q., Wan X., Zhao J., Cheng Q. (2014). Coding metasurfaces, digital metasurfaces and programmable metasurfaces. Light Sci. Appl..

[7] Foschini G.J. (1996). Layered space-time architecture for wireless communication in a fading environment. Bell Labs Tech. J..

[8] Telatar I.E. (1999). Capacity of multi-antenna Gaussian channels. Eur. Trans. Telecommun..

[9] Paulraj A., Nabar R., Gore D. (2003). Introduction to Space-Time Wireless Communications.

[10] Blanch S., Romeu J., Corbella I. (2003). Exact representation of antenna system diversity performance from input parameter description. Electron. Lett..

[11] Chiu C.-Y., Cheng C.-H., Murch R.D., Rowell C.R. (2007). Reduction of mutual coupling between closely-packed antenna elements. IEEE Trans. Antennas Propag..

[12] Kumar S., Singh H. (2022). A comprehensive review of metamaterials/metasurface-based MIMO antenna array for 5G millimeter-wave applications. J. Supercond. Nov. Magn..

[13] Pannu P., Sharma D.K. (2020). A low-profile quad-port UWB MIMO antenna using defected ground structure with dual notch-band behavior. Int. J. RF Microw. Comput.-Aided Eng..

[14] Ren A., Yu H., Yang L., Huang Z., Zhang Z., Liu Y. (2023). A broadband MIMO antenna based on multimodes for 5G smartphone applications. IEEE Antennas Wirel. Propag. Lett..

[15] Swain J., Mohanty A., Behera S.K., Bhowmik W. (2025). Metasurface Loaded Closely Spaced Shared Radiator 4-port MIMO Antenna with Dual-Radiation Performance. 2025 International Conference on Microwave, Optical, and Communication Engineering (ICMOCE).

[16] Huang J., Encinar J.A. (2008). Reflectarray Antennas.

[17] Balanis C.A. (2016). Antenna Theory: Analysis and Design.

[18] Mailloux R.J. (2005). Phased Array Antenna Handbook.

